# Overexpression of *BnPCS1*, a Novel Phytochelatin Synthase Gene From Ramie (*Boehmeria nivea*), Enhanced Cd Tolerance, Accumulation, and Translocation in *Arabidopsis thaliana*

**DOI:** 10.3389/fpls.2021.639189

**Published:** 2021-06-15

**Authors:** Shoujing Zhu, Wenjuan Shi, Yucheng Jie

**Affiliations:** ^1^College of Life Sciences, Resources and Environment, Yichun University, Yichun, China; ^2^Key Laboratory of Crop Growth and Development Regulation of Jiangxi Province, Yichun, China; ^3^Institute of Ramie, Hunan Agricultural University, Changsha, China

**Keywords:** *Boehmeria nivea*, phytochelatin synthase, cadmium, accumulation, translocation

## Abstract

Phytochelatins (PCs) play important roles in the detoxification of and tolerance to heavy metals in plants. The synthesis of PCs is catalyzed by phytochelatin synthase (PCS), which is activated by heavy metal ions. In this study, we isolated a *PCS* gene, *BnPCS1*, from the bast fiber crop ramie (*Boehmeria nivea*) using the RACE (rapid amplification of cDNA ends) method. The full-length *BnPCS1* cDNA is 1,949 bp in length with a 1,518 bp open reading frame (ORF) that encodes a 505 amino acid protein. The deduced BnPCS1 protein has a conserved N-terminus containing the catalytic triad Cys^58^, His^164^, Asp^182^, and a flexible C-terminal region containing a C^371^C^372^QETC^376^VKC^379^ motif. The *BnPCS1* promoter region contains several cis-acting elements involved in phytohormone or abiotic stress responses. Subcellular localization analysis indicates that the BnPCS1-GFP protein localizes to the nucleus and the cytoplasm. Real-time PCR assays show that the expression of *BnPCS1* is significantly induced by cadmium (Cd) and the plant hormone abscisic acid (ABA). Overexpression lines of *BnPCS1* exhibited better root growth and fresh weight, lower level of MDA and H_2_O_2_, and higher Cd accumulation and translocation factor compared to the WT under Cd stress. Taken together, these results could provide new gene resources for phytoremediation of Cd-contaminated soils.

## Highlights

- The expression of *BnPCS1* is significantly induced by Cd and ABA.
- Overexpression of *BnPCS1* confers enhanced Cd tolerance, accumulation and translocation in Arabidopsis.
- Our research results could provide new gene resources for phytoremediation.

## Introduction

Heavy metal pollution has been recognized one of the most important environmental issues worldwide. When the concentrations of heavy metal ions in soils reach a certain level, plants growing in these soils will exhibit various symptoms of poisoning, including growth retardation, stunting, chlorosis, and finally growth cessation. However, plants have developed diverse adaptive mechanisms during the course of evolution, and some plants display stronger tolerance to the effects of heavy metals (Emamverdian et al., 2015). Phytochelatins (PCs) are small thiolate peptides produced immediately after exposure to heavy metal ions such as Cd^2+^, As^2+^, Pb^2+^, Cu^2+^, and Zn^2+^, that exist widely in certain bacteria, algae, fungi, and almost all plant species. PCs have strong chelation activity and play important roles in the accumulation and detoxication of heavy metal ions in plants by chelating these toxic ions to form complexes. The complexes are then sequestered to specific organelles (mainly to the vacuoles) and fixed by a process known as compartmentalization so that the concentration of toxic metal ions will be reduced to levels that plants can tolerate (Kim et al., 2019).

PCs have the general structure (γ-Glu-Cys)_*n*_-Gly (*n* = 2–11) and are synthesized from glutathione (GSH, γ-Glu-Cys-Gly), catalyzed by phytochelatin synthase (PCS, EC 2.3.2.15) (Ramos et al., 2007). PCS belongs to the papain superfamily; it is actually a dipeptidyl transferase in which the catalytic reaction process is similar to that of cysteine protease. The synthesis of PCs can be divided into two stages: in the first stage, the γ-Glu-Cys unit from GSH is cleaved and transferred to the free enzyme to generate a kind of acyl-enzyme intermediate. In the second stage, the γ-Glu-Cys unit is transferred from the intermediate to another substrate molecule which can be GSH or an oligomeric phytochelatin peptide (PC_*n*_) to form PC_*n*__+__1_. The cycle is then repeated until the final products (γ-Glu-Cys)_2__–__11_-Gly, are produced (Vatamaniuk et al., 2004). The reactions are shown below (Rigouin et al., 2013):

Stage 1: γ−Glu−Cys−Gly+PCS→γ−Glu−Cys−PCS+Gly

Stage 2: γ−Glu−Cys−PCS+(γ−*Glu*−*Cys*)_*n*_Gly→PCS+(γ−*Glu*−*Cys*)_*n*+1_−Gly

In the fission yeast (*Schizosaccharomyces pombe*) and various plant species, the amino acid sequences of most eukaryotic PCSs consist of conserved N-terminal and variable C-terminal domains. The N-terminal domains from most organisms share high levels of sequence homology, and are suggested to have catalytic activity (Hayashi et al., 2020). There are several conserved Cys residues in the N-terminal domains, among which the Cys^57^ residue is present in almost all known PCS proteins, and it may be related to the catalytic activity of PCS enzymes. The C-terminal domain is considered to function in regulating metal ion activity, and contains pairs of Cys and Glu residues. In the absence of heavy metals, the N-terminal domains have no enzymatic activity. When heavy metals are detected, the C-terminal domain forms a special structure with the heavy metal ions that can initiate catalytic activity in the N-terminal domain. The ability of different metal ions to induce catalytic activity in PCS enzymes varies, and Cd^2+^ has been shown to be the most efficient metal catalyst (Filiz et al., 2019).

A PCS enzyme with biological activity was first isolated from *Silene cucubalus* in 1989 (Grill et al., 1989), followed by similar reports from *Arabidopsis thaliana* (Vatamaniuk et al., 1999), *Pteris vittata* (Dong, 2005), *Sesbania rostrata* (Li et al., 2009), *Triticum aestivum* (Couselo et al., 2010), tall fescue (Zhao et al., 2014) *Oryza sativa* (Das et al., 2017), and *Ipomoea pes-caprae* (Su et al., 2020). *AtPCS1*-deficient Arabidopsis plants are highly sensitive to Cd, while overexpression of *AtPCS1* changed the Cd tolerance and the ability of plants to accumulate Cd (Ha et al., 1999; Pomponi et al., 2006). Tobacco plants expressing *NtPCS1* showed an increased tolerance to arsenic (As) and Cd, but changes in the accumulation of As and Cd were not observed (Lee and Hwang, 2015). Heterologous expression of the *CdPCS1* gene from *Ceratophyllum demersum* in Arabidopsis and *Escherichia coli* enhanced the accumulation of heavy metals (Shukla et al., 2012).

*Boehmeria nivea* (L.) Gaudich., commonly known as ramie, is a perennial herb in the nettle family (the *Urticaceae*) that is native to eastern Asia. Ramie is also known as “China grass,” and the fibers are widely used in the textile industry to make fabric. Plants of *B. nivea* have well-developed root systems, fast growth, high reproducibility, strong resistance to stress and disease, and produce a large amount of biomass, characteristics that can quickly make up for the shortages of other known heavy metal hyperaccumulators. In addition, ramie is used as a raw industrial material that will not enter the food chain and can produce economic benefits. Previous studies have shown that ramie has strong heavy metal tolerance and the ability to accumulate several heavy metals from soil (She et al., 2011; Shukla et al., 2012). However, the mechanisms that determine Cd tolerance in ramie are unclear. In our previous studies on the transcriptome profiles of Cd-responsive genes in ramie, unigene6921 was found to be significantly up-regulated in the Cd treatment group, and this gene was annotated as a phytochelatin synthase gene (She et al., 2015). In this paper, we isolated the full-length cDNA of the PCS gene unigene6921 using the RACE method (rapid amplification of cDNA ends). The gene is designated *BnPCS1*, and our study focused on gene promoter analysis, subcellular localization of the BnPCS1 protein, gene expression characteristics, and the possible functions of *BnPCS1* in the response to Cd stress in *B. nivea*.

## Materials and Methods

### Plant Growth Conditions and Hormone and Cd Treatments

The *B. nivea* cultivar “Zhongzhu No. 1” used as plant material in this study was obtained from the Yunyuan farm of Hunan Agricultural University, Changsha, China. The ramie seedlings were grown hydroponically in half-strength Hoagland nutrient solution for 21 days. For the hormone treatments, the seedlings were sprayed with 100 μM abscisic acid (ABA) or 1 mM salicylic acid (SA). For the Cd treatment, seedlings were transferred to half-strength Hoagland solution containing 200 μM Cd. Roots, stems, and leaves were harvested after 0, 2, 4, 6, 12, and 24 h of ABA or SA treatment. Leaves were harvested after 0, 3, 6, 9, 12, 24, and 48 h of Cd treatment. The roots and shoots were frozen immediately in liquid nitrogen and stored at −80°C. For each time point there were three biological replicates.

### Isolation of RNA and Genomic DNA and First-Strand cDNA Synthesis

Total RNA was extracted from plant tissues using the RNAprep pure Plant Kit (Tiangen, Beijing, China). For first-strand cDNA synthesis, ∼1.0 μg of total RNA was used as the template in the PrimeScript RT reagent Kit with gDNA Eraser Kit (Takara, Beijing, China) following the manufacturer’s instructions. Genomic DNA was extracted from *B. nivea* tissues using the Rapid Plant Genomic DNA Isolation Kit (Shanghai Shenggong Co., Shanghai, China) as directed by the manufacturer.

### Isolation of the Full-Length *BnPCS1* cDNA

To obtain the full-length cDNA sequence of *BnPCS1*, the 5′RACE primers PCS1-5FO/5FI and the 3′RACE primers PCS1-3FO/3FI (Supplementary Table 1) were designed based on the sequence of unigene6921 from the ramie transcriptome using Prime Premier 5.0 software. The 5′RACE and 3′RACE reactions were performed using the SMARTer RACE5′/3′ Kit (Clontech, United States) following the manufacturer’s protocol. The products from 5′RACE and 3′RACE were examined on 1.5% agarose gels, purified with the TaKaRa MiniBEST Agarose Gel DNA Extraction Kit (Takara, Beijing, China), and cloned into the pMD18-T vector (Takara, Beijing, China) prior to DNA sequencing. The sequences from 5′ RACE, 3′RACE, and the known gene fragment were then assembled using DNAMAN 8.0 software. To verify the accuracy of the assembled sequence, a pair of primers PCS1-F/R (Supplementary Table 1) was used to amply the full-length sequence which was then sequenced at Shanghai Shenggong Company (Shanghai, China). The nucleotide sequence of *BnPCS1* was submitted to the National Center for Biotechnology Information (NCBI) Genbank database under Accession No. KF717368.

### Isolation of the *BnPCS1* Gene Promoter

Genomic DNA extracted from ramie cultivar “Zhongzhu No. 1” was used as the template to amplify the 2,057 bp of genomic DNA sequence upstream from the *BnPCS1* initiation codon using the primer pair PCS1-PF/PR (Supplementary Table 1). The PCR amplification proceeded for 32 cycles of denaturation at 98°C for 10 s, primer annealing at 55°C for 15 s, and extension at 72°C for 30 s using PrimeSTAR Max DNA Polymerase (Takara, Beijing, China). The PCR product was examined by agarose gel electrophoresis, purified, and sequenced. The cis-acting regulatory elements in the *BnPCS1* promoter region were predicted by searching the PlantCARE database^1^.

### Sequence Analysis

The *BnPCS1* ORF was analyzed and translated with the ORF Finder^2^. The physicochemical properties of the amino acid sequences deduced from *BnPCS1* were predicted with the ProtParam tool on the ExPASy server^3^. The sequence comparison was performed using the BLASTP tool^4^. The conserved domains in the BnPCS1protein were predicted with the Conserved Domain Search Service (CD Search)^5^. Multiple sequence alignment was performed with DNAMAN 8.0 software. A phylogenetic tree showing the evolutionary relationships between BnPCS1 and predicted PCS proteins from other plant species was constructed with MEGA 5.0 software using the neighbor-joining (NJ) method based on Kimura’s 2-parameter distance and 1,000 bootstrap replicates to estimate the confidence of the individual branches. The protein subcellular localization was predicted withWoLF PSORT^6^.

### Subcellular Localization of *BnPCS1* Protein

A pair of specific primers, PCS1-XbaI-F and PCS1-SmaI-R (Supplementary Table 1), was used to amplify the *BnPCS1* ORF. The PCR product was digested with the restriction enzymes XbaI and SmaI and cloned into the expression vector pAN580, so that the BnPCS1 protein is fused to the green fluorescent protein (GFP) reporter gene toconstruct plasmid 35S-BnPCS1-GFP. The empty vector 35S::GFP was used as the control while the 35S::OsGhd7-CFP plasmid was used to produce the nuclear marker. The constructs were separately introduced into Arabidopsis protoplasts. Protoplast isolation and transfection were performed according to the method described by Yoo et al. (2007). Briefly, mesophyll protoplasts were isolated from the rosette leaves collected from 3 to 4-weeks-old Col wild type *Arabidopsis thaliana* plants. Then, the nuclear marker construct 35S::OsGhd7-CFP was co-transformed with 35S-BnPCS1-GFP constructor 35S::GFP construct into *Arabidopsis* protoplasts. The fluorescence signals of GFP and CFP (cyan fluorescentprotein) were observed with a confocal microscope imaging system (Olympus FV1200) after the protoplasts were incubated at room temperature for 20–22 h in the dark.

### Real-Time Quantitative RT-PCR Analysis

Real-time quantitative RT-PCR (qRT-PCR) was performed using the StepOnePlus Real-Time PCR System (Applied Biosystems). The ramie actin gene (DQ665832) was amplified along with *BnPCS1* as a reference gene to normalize gene expression. For tissue-specific expression analysis, total RNA was extracted from the roots, stems, stem tips, and leaves of ramie seedlings. First-strand cDNA synthesis was carried out using the PrimeScript RT reagent kit with gDNA Eraser (TaKaRa, Beijing, China) following the manufacturer’s instructions. For gene expression analysis of plants treated with Cd, cDNA was synthesized from total RNA extracted from roots, stems, and leaves of seedlings treated with Cd solution at different time points. The nucleotide sequences of the primers used to amplify *BnPCS1* (PCS1-qF/R) and the actin gene (BnActin-F/R) are given in Supplementary Table 1. Quantification of the relative transcript levels was performed using the comparative 2^–ΔΔ*Ct*^ method (Livak and Schmittgen, 2001). The qRT-PCR assays were conducted with three biological replicates and three independent technical replicates for each sample.

### Over-Expression of *BnPCS1* in Arabidopsis

To over-express *BnPCS1* in Arabidopsis, the complete *BnPCS1* ORF was amplified by PCR with a pair of gene-specific primers with added 5’ restriction enzyme sites (PCS1-SmaI-F and PCS1-BamHI-R). The PCR amplification proceeded for 32 cycles and consisted of denaturation at 98°C for 10 s, primer annealing at 60°C for 15 s, and extension at 72°C for 30 s. After double-digestion with restriction enzymes SmaI and BamHI, the PCR-amplified product was subcloned into the plant expression vector pBI121 to make the construct named pBI121-BnPCS1. The recombinant plasmid was introduced into the *Agrobacterium tumefaciens* strain EHA105 using the freeze-thaw method, and the construct was then transformed into Arabidopsis Col-0 plants using the floral-dip method (Clough and Bent, 1998; Zhang et al., 2006). Briefly, *Agrobacterium* cultures were grown on a platform shaker (220 r.p.m.) at 28°C to stationary phase in sterilized LB medium (containing 10 g tryptone, 5 g NaCl, 5 g yeast extract per liter water) carrying added kanamycin (30 mg⋅L^–1^). Cultures were typically started from a 1:100 dilution of smaller overnight cultures and grown for roughly 18–24 h. Cells were harvested by centrifugation at 5,500 × g for 20 min and resuspended in infiltration medium (containing 5% sucrose, 0.05 % Silwet L-77, 2.15 g⋅L^–1^ MSsalts) to a final OD_600_ = 0.8. Then, the medium was added to a beaker, plants were inverted into this medium such that all above-ground parts were submerged, and plants were removed after 3–5 s of gentle agitation. Plants were removed from the beaker, domed to remain humidity and covered with black cloth. The cloth was removed the next day and plants were regularly watered until seeds maturation. Seeds (T_3_ generation) of the homozygous transgenic lines were screened on 1/2 strength MS medium containing 30 mg⋅L^–1^ kanamycin. The kanamycin resistant lines were further verified for the presence of the *BnPCS1* gene by PCR and real-time RT-PCR. The primers PCS1-35S-F and PCS1-SP-R used for PCR verification are given in Supplementary Table 1.

### Cd Stress Assay

#### Phenotypic Analysis

To test the effect of Cd stress on the growth of transgenic Arabidopsis seedlings, seeds from the transgenic lines (T_3_ generation) and wild-type (WT) plants were cultured on half-strength solid Murashige and Skoog medium (per liter add 100 ml 10 × macronutrients, 10 ml 100 × micronutrients, 5 ml 1% Fe-EDTA, 30 g sucrose, pH 5.6–5.8, with 1 M KOH, 7 g agar) (Murashige and Skoog, 1962) containing Cd (0, 100, or 150 μM). The fresh weights and root lengths of transgenic and wild-type Arabidopsis seedlings were measured at 14 days, and the plants were photographed. WT seedlings were used as the control. There were three experimental replicates.

#### Determination the Cd Content in Plants

The transgenic and wild-type Arabidopsis were planted in plastics pots with unpolluted vermiculite and irrigated with half-strength Hoagland nutrient solution containing 2.5 mM Ca(NO_3_)_2_, 2.5 mM KNO_3_, 1 mM MgSO_4_, 1 mM KH_2_PO_4_, and 23.1 μM H_3_BO_3_, 4.6 μM MnCl_2_, 0.19 μM CuSO_4_, 1.2 μM ZnSO_4_, 0.12 μM Na_2_MoO_4_ and 45 μM Fe(III)-EDTA at pH 6.0 (Terry, 1980) for 28 days. Then, CdCl_2_ salt was added to the half-strength Hoagland nutrient solution to the final concentration of 50 μM, and the plants were watered with the Cd solution one time. After 14 days of cultivation, the plants were harvested, washed with tap water and rinsed with deionized water three times. Then, the shoots and roots were separated and dried to a constant weight (65°C for 72 h). After that, Cd in the samples was measured by flame AAS with HNO_3_-HClO_4_ digestion. There were three experimental replicates.

Translocation factor (TF) is calculated from the ratio of cadmium’s presence in the plant shoots compared to that in roots using the equation:

$$\text{TF}=\frac{\mathrm{Cd}\,\left(\mathrm{plant}\,\mathrm{shoot}\right)}{\mathrm{Cd}\,\left(\mathrm{plant}\,\mathrm{root}\right)}$$

#### Determining the H_2_O_2_ Content

Twenty-one-day-old seedlings from the transgenic lines (T_3_ generation) and WT cultured in soil without Cd were irrigated with 0, 100, or 150 μM Cd solution and grown for another 2 days. The H_2_O_2_ content was determined using the method described by DeLong et al. (2002). Briefly, a 1-gram sample of leaf tissue was ground into a powder and extracted with 80 percent ethanol. The homogenate was then centrifuged at 10,000 × g for 10 min, and 0.1 mL of the supernatant was added to 1 mL working solution (containing 100 μM xylenol orange, 250 μM ammonium ferrous sulfate hexahydrate, 90 percent methanol, and 25 mM H_2_SO_4_) and incubated for 30 min at 30°C. The absorbance of the reaction mixtures was measured at 560 nm with a UV-visible spectrophotometer (UV-1800, Shimadzu, Japan). The standard curve was established using H_2_O_2_ concentrations ranging from 0 to 100 μM to calculate the content of H_2_O_2_ in the unknown samples.

#### Determining the MDA Content

Twenty-one-day-old seedlings from the transgenic lines (T_3_ generation) and WT plants cultured in the soil without Cd were irrigated with 0, 100, or 150 μM Cd solution and further grown for 14 days. The content of MDA was determined according to the method described by Hodges et al. (1999). Leaf tissue samples (0.5 g) were ground to powder and extracted with 10 mL of 20 percent trichloroacetic acid (TCA). The homogenates were centrifuged at 10,000 × g for 10 min, and 2 mL of supernatant from each extract was added to 2 mL of 0.6 percent thiobarbituric acid (TBA) solution containing 10 percent TCA and incubated at 100°C for 15 min. The reaction mixtures were then rapidly cooled in an ice bath. The absorbance of each supernatant was measured at 450, 532, and 600 nm using a UV-Visible spectrophotometer (UV-1800, Shimadzu, Japan). The MDA content was calculated as 6.45 × (A_532_ - A_600_) - 0.56 × A_450_.

### Statistical Analysis

Statistical analyses were conducted using SPSS version 17.0 and Microsoft Excel 2013 software. All data were expressed as the mean of three biological replicates ± standard deviation (SD). Comparisons between different groups were tested by one-way ANOVA followed by Student’s *t*-test. A *p*-value < 0.05 was considered to indicate a significant difference.

## Results

### Isolation and Characterization of *BnPCS1*

The 1,949 bp full-length cDNA for the *BnPCS1* gene was isolated using 3′-RACE, 5′-RACE, and RT-PCR methods. DNA sequence analysis showed that the cDNA contains a 1,518 bp open reading frame (ORF) that is predicted to encode a 505 amino acid protein (Supplementary Figure 1). The physicochemical properties of the predicted BnPCS1 protein were analyzed using the ExPASy database, which showed that the BnPCS1 protein has a predicted molecular mass of 56.02 kDa and an isoelectric point of 7.01. The secondary structure of the BnPCS1 protein was predicted using the NPS@(Network Protein Sequence Analysis) server, which indicated that the BnPCS1 protein consists of α-helices (49.70%), extended strands (11.49%), and random coils (38.81%). The protein subcellular localization predicted using WoLFPSORT showed that BnPCS1 protein is mainly located in both nucleus and cytoplasm.

**FIGURE 1:**
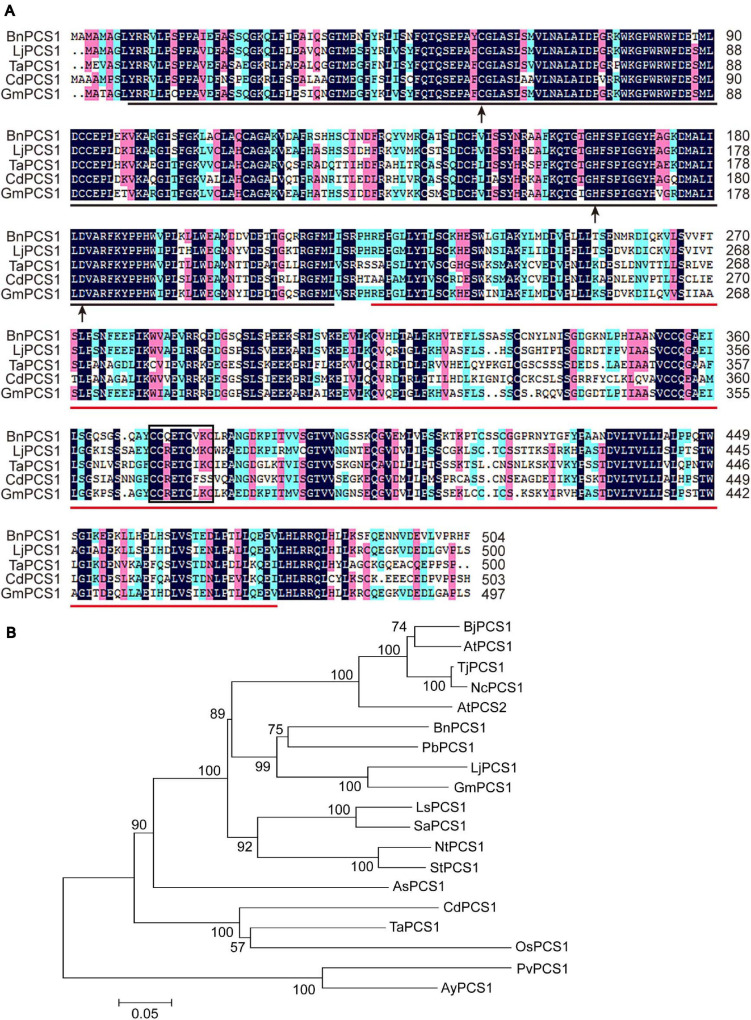
Multiple sequence alignment and phylogenetic analysis of *BnPCS1* and PCS proteins from other plant species. **(A)** Alignment of *BnPCS1* and its orthologous proteins from various plant species constructed using DNAMAN 8.0 software. The shaded regions indicate the conserved amino acid residues (those shown in black are fully conserved; the amino acids highlighted in pink and light green are similar). The region underlined in black is the phytochetatin domain, and the phytochelatin_C domain is underlined in red. The three active sites are indicated by black arrows. The C^371^C^372^QETC^376^VKC^379^ motif is enclosed in a black box. **(B)** Phylogenetic tree showing the evolutionary relationships between *BnPCS1* and 18 other PCS proteins from various plant species constructed using the neighbor-joining (NJ) method in MEGA 5.0. The scale bar represents 0.05 amino acid substitutions per site. GenBank accession numbers of the proteins are as follows: AtPCS1 (AAD16046.1) and AtPCS2 (AAK94671.1) from *Arabidopsis thaliana*, BjPCS1 (CAC37692.1) from *Brassica juncea*, TjPCS1 (BAB93119.1) from *Thlaspi japonicum*, NcPCS1 (BAB93120.1) from *Noccaea caerulescens*, PbPCS1 (AEY68568.1) from *Pyrus betulifolia*, LjPCS1 (AAT80342.1) from *Lotus japonicus*, GmPCS1 (AAL78384.1) from *Glycine max*, LsPCS1 (AAU93349.1) from *Lactuca sativa*, SaPCS1 (ACU44656.1) from *Sonchus arvensis*, NtPCS1 (AAO74500.1) from *Nicotiana tabacum*, StPCS1 (CAD68110.1) from *Solanum tuberosum*, AsPCS1 (AAO13809.1) from *Allium sativum*, CdPCS1 (AAO13810.2) from *Cynodon dactylon*, TaPCS1 (AAD50592.1) from *Triticum aestivum*, OsPCS1 (AAO13349.2) from *Oryza sativa*, and PvPCS1 (AAT11885.1) and AyPCS1 (BAB64932.1) from the ferns *Pteris vittata* and *Athyrium yokoscense*, respectively.

A conserved domain search of the NCBI database showed that the deduced amino acid sequence of BnPCS1 contains two domains: a phytochetatin domain at amino acids 9–217 and a phytochelatin_C domain at amino acids 223–478. There are three active sites in the phytochetatin domain; (1) the cysteine residue at amino acid residue 58, (2) the histidine residue atamino acid residue 164, and (3) the aspartic acid residue at amino acid residue 182. A C^371^C^372^QETC^376^VKC^379^ motif in the PCS serves as a sensor for heavy metal ions and was found within the phytochelatin_C domain (Figure 1A). BLASTp searches and multiple sequence alignments revealed that the BnPCS1 protein shares a high degree of sequence similarity with the reported phytochelatin synthases from other plant species such as *Lotus japonicus* (GenBank accession number: AAT80342.1; 73%), *Triticum aestivum* (AAD50592.1; 61%), *Cynodon dactylon* (AAO13810.2; 57%), and *Glycine max* (AAL78384.1; 73%). In addition, the N-terminal part of the PCS proteins is more conserved than the C-terminal part (Figure 1A).

To analyze the evolutionary relationships of PCS proteins, the sequences of BnPCS1 and 18 previously reported PCS proteins from various plant species were downloaded from GenBank and used to construct a phylogenetic tree using the neighbor-joining method as implemented in MEGA 5.0 software (Tamura et al., 2007). The phylogenetic tree showed that BnPCS1 has the closest evolutionary relationship with PbPCS1, LjPCS1, and GmPCS1, which come from dicotyledonous plants in the order Rosales. BnPCS1 is more distantly related to CdPCS1, TaPCS1, OsPCS1, PvPCS1, and AyPCS1, which belong to two clades of PCS1 proteins from monocotyledonous plants (Figure 1B).

### Isolation of the *BnPCS1* Promoter

A pair of primers, PCS1-PF and PCS1-PR (Supplementary Table 1), were designed to isolate a 1,928 bp DNA fragment containing the *BnPCS1* promoter (Supplementary Figure 2). Analysis ofthe cis-acting elements in the promoter sequence using searches of the PlantCARE database indicated that the promoter contains not only the basic promoter elements such as the CAAT-box and TATA-box, but also some cis-acting elements associted with stress responses, such as an anaerobic induction element (ARE), a drought responsive element (DRE1), a stress responsive element (STRE), and a wound responsive element (WUN-motif). Also present were some hormone response elements, such as an abscisic acid responsive element (ABRE), an MeJA responsive element (CGTCA-motif), a salicylic acid responsiveelement (TCA-element), and an auxin responsive element (TGA-element) (Table 1). These results suggest that *BnPCS1* may participate in responses to abiotic and biotic stresses.

**TABLE 1:** Putative cis-acting regulatory elements identified inthe promoter region sequence of *BnPCS1* using thePlantCARE database.

Cis element	Position	Sequence	Function of site
ABRE	+717	ACGTG	Cis-acting element involved in the abscisic acid responsiveness
ARE	+235	AAACCA	Cis-acting regulatory element essential for the anaerobic induction
CAAT-box	+122, -220, +316, +440, +615, +861, +938, +1018, +1131, -1179, +1253, +1360, -1409, +1787	CAAT, CAAAT, CCCAATTT, CCAAT	Common cis-acting element in promoter and enhancer regions
CGTCA-motif	-427	CGTCA	Cis-acting regulatory element involved in the MeJA-responsiveness
DRE1	-1725	ACCGAGA	Cis-acting regulatory element involved in the drought responsiveness
G-box	+47, +716	CACGAC, TACGTG	Cis-acting regulatory element involved in light responsiveness
Gap-box	+440	CAAATGAAAA	Part of a light responsive element
MRE	+172	AACCTAA	MYB binding site involved in light responsiveness
STRE	+594, +1150, -1122	AGGGG	Cis-acting regulatory element involved in stress responsiveness
TATA-box	-241, +271, -290, -323, -325, +342, -347, +355, -481, +484, -509, -525, -568, -623, +627, +702, +706, +941, -976, -1025, -1030, +1077, -1090, -1210, -1220, -1241, -1270, -1413, -1599, -1623, -1627	TACAAAA, TATTTAAA, TACATAAA, TATAAAT, TATATAA, TATAA,TATA, TATATA, TATAT, TATAAAA	Core promoter element around -30 of transcription start
TCA-element	-603, +1008	CCATCTTTTT	Cis-acting element involved in salicylic acid responsiveness
TGA-element	-1501, -1748	AACGAC	Auxin-responsive element
WUN-motif	-1318, -1351, -1319	AAATTACTA, AAATTACT	Cis-acting regulatory element involved in the wound responsiveness
Circadian	+778	CAAAGATATC	Cis-acting regulatory element involved in circadian control

### Subcellular Localization of the *BnPCS1* Protein

**FIGURE 2:**
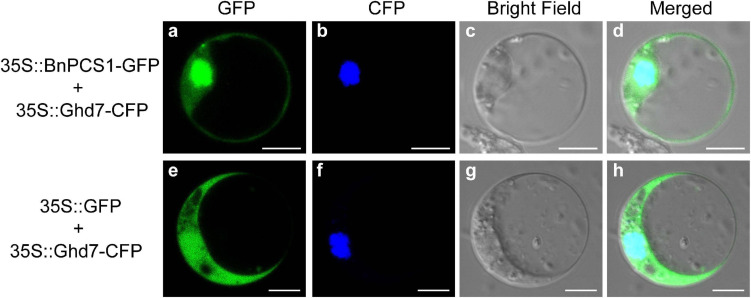
Subcellular localization of BnPCS1. Recombinant plasmid 35S::BnPCS1-GFP and the empty vector 35S::GFP were transfected separately into Arabidopsis protoplasts with nuclear marker 35S::OsGhd7-CFP. The CFP and GFP fluorescence signals were observed using a laser confocal microscope. **(a)** 35S::BnPCS1-GFP; **(b)** 35S::OsGhd7-CFP; **(c)** bright field; **(d)** overlap images of **(a–c)**; **(e)** 35S::GFP; **(f)** 35S::OsGhd7-CFP; **(g)** bright field; **(h)** overlap images of **(e–g)**.

The protein subcellular localization predicted using WoLF PSORT indicated that BnPCS1 protein is mainly localized in both nucleus and cytoplasm. To verify this prediction, the recombinant plasmid 35S::BnPCS1-GFP, which contains an enhanced GFP gene fused to *BnPCS1* in pAN580, was transfected into Arabidopsis protoplasts, the empty 35S::GFP vector wasused as the control. Microscopic observation showed that the green fluorescence in cells transfected with 35S::BnPCS1-GFP were observed both in the nucleus and the cytoplasm along with the cyan fluorescent signal of nuclear marker OsGhd7-CFP (Figures 2a–d). In contrast, green fluorescence was detected throughout the cells expressing the GFP (Figures 2e–h) gene alone. The above results indicated that BnPCS1 protein is localizedin both the nucleus and cytoplasm of plant cells.

### Gene Expression Analysis of *BnPCS1* in *B. nivea*

**FIGURE 3:**
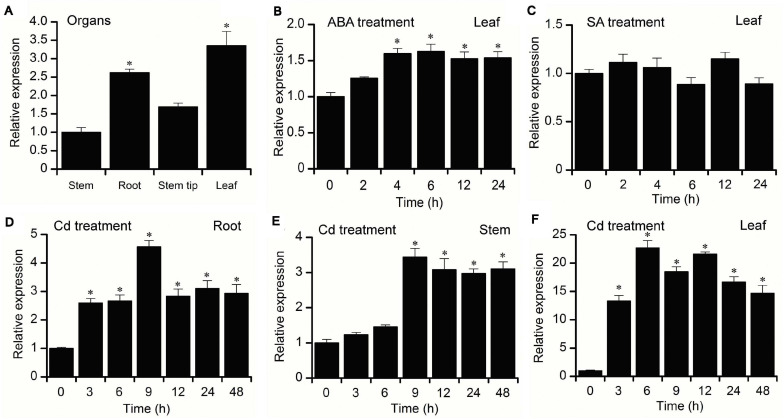
Expression pattern of *BnPCS1*. **(A)** qRT-PCR analysis of *BnPCS1* mRNA levels in the roots, stems, stem tips, and leaves of 21-day-old *B. nivea* seedlings. **(B,C)** *BnPCS1* expression levels in the leaves of *B. nivea* seedlings treated with 100 μM ABA **(B)** or 1 mM SA **(C)** for 0, 2, 4, 6, 12, and 24 h. **(D–F)**: *BnPCS1* expression levels in the roots **(D)**,stems **(E)**, and leaves **(F)** of *B. nivea* seedlings treated with 200 μM Cd for 0, 3, 6, 9, 12, 24, and 48 h. Data are presented as the means of three biological replicates with SE shown by vertical bars. Asterisks indicate significant differences (*p* < 0.05) between the treatment groups and the controls.

To examine the tissue-specific expression characteristics of *BnPCS1*, the transcriptional levels of *BnPCS1* in the root, stems, stem tip, and leaves of 21-day-old *B. nivea* seedlings were analyzed using quantitative real-time PCR (qRT-PCR). The results showed that the highest expression level of *BnPCS1* was in leaves, and the lowest expression level was in the stems (Figure 3A). For the hormone treatments, the 21-day-old *B. nivea* seedlings were sprayed with 100 μM abscisic acid (ABA) or 1 mM salicylic acid (SA), respectively. Expressions of *BnPCS1* in *B. nivea* leaves after treatment with ABA or SA for 0, 2, 4, 6, 12 or 24 h were assayed by qRT-PCR. We found that the relative expression of *BnPCS1* is rapidly induced by ABA and reached its highest level after 6 h, which was 1.6-fold the level in untreated leaves (Figure 3B). However, the expression of *BnPCS1* was not significantly induced by SA treatment (Figure 3C). To investigate the effects of Cd treatment on the expression of *BnPCS1*, the mRNA levels of *BnPC*S*1* in *B. nivea* were examined by qRT-PCR after treatment with 200 μM Cd for 0, 3, 6, 9, 12, 24, or 48 h. The results indicated that *BnPCS1* is significantly up-regulated by Cd treatment in the roots, stems, and leaves of *B. nivea* seedlings. The highest expression levels of *BnPC*S*1* in the roots, stems, and leaves were 4. 6-, 3. 4-, and 22.7-fold higher than in the untreated samples, respectively (Figures 3D–F).

### Overexpression of *BnPCS1* Enhanced Cd Tolerance and Accumulation in Transgenic Arabidopsis Plants

To investigate whether *BnPCS1* is involved in the response of plants to Cd, the complete coding region of *BnPCS1* was cloned and inserted into the binary vector pBI121 to generate the pBI121-BnPCS1 construct in which *BnPCS1* was over-expressed under control of the CaMV 35S promoter. The recombinant plasmid in *Agrobacterium tumefaciens* strain EHA105 was then transformed into Arabidopsis ecotype Columbia (Col-0) using the floral-dip method. Seeds obtained from the T_3_ generation of transgenic Arabidopsis plants were screened on half-strength MS medium supplemented with 30 mg⋅L^–1^ kanamycin (Supplementary Figure 3). Of these, five transgenic lines (designated L1, L2, L3, L4, and L5) were found to be positive for *BnPCS1* following PCR screening (Supplementary Figure 4). Real-time PCR analysis showed that the *BnPCS1*-specific mRNA levels in the five transgenic Arabidopsis lines varied, and *BnPCS1* expression was not detected in WT plants. The highest mRNA level was found in line L1, followed by line L3, and both lines were selected for additional analysis (Supplementary Figure 5).

**FIGURE 4:**
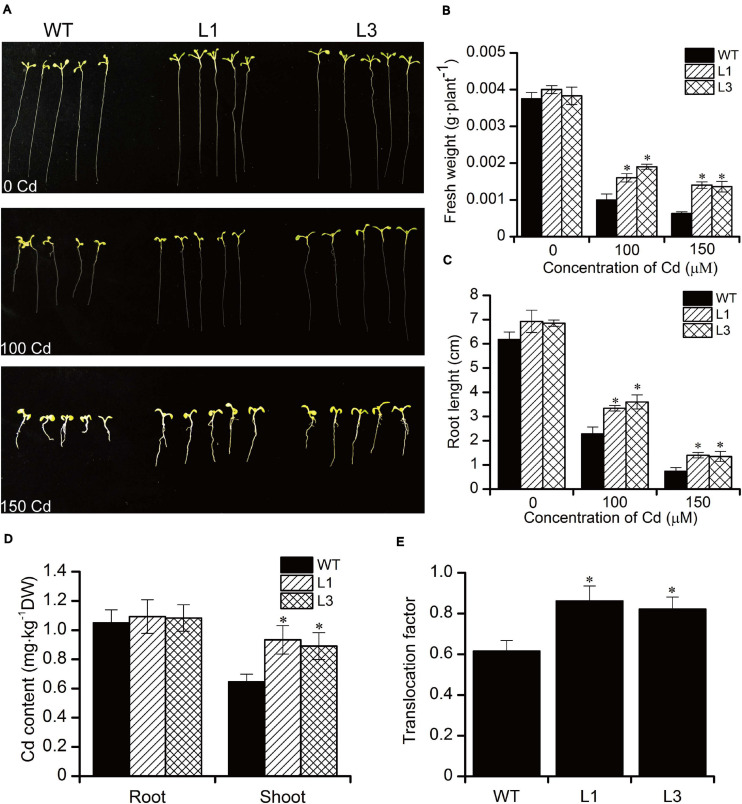
Analysis of the response to Cd exposure in WT and *BnPCS1* transgenic Arabidopsis plants. **(A)** Root lengths in *Arabidopsis* seedlings grown on 0.5× MS medium containing 0, 100, or 150 μM Cd for 14 days. **(B,C)** Analysis of fresh weights and root lengths of *Arabidopsis* seedlings grown on 0.5× MS medium containing 0, 100, or 150 μM Cd for 14 days. **(D)** Cd content in roots and shoots of WT and transgenic *Arabidopsis* plants grown in vermiculite irrigated with 50 μM Cd for 14 days; **(E)** Translocation factor (TF) of Cd in WT, L1 and L3. Data are presented as the means of three biological replicates with SE shown by vertical bars. Asterisks indicate significant differences (*p* < 0.05) between the transgenic lines compared to WT.

To analyze the effect of Cd stress on the growth of *BnPCS1*-expressing transgenic Arabidopsis lines (L1 and L3)as compared to WT, seeds of the T_3_ generation from WT, L1, and L3 were germinated and grown on half-strength MS medium containing three concentrations of Cd (0, 100, and 150 μM). The fresh weights and root lengths of transgenic and wild-type *Arabidopsis* seedlings were measured at 14 days. There were no significant differences in the phenotypes and growth between the transgenic and WT plants under normal conditions without Cd in the medium. Following Cd treatment, the growth of all plants was inhibited (Figure 4A). However, the fresh weights and root lengths of the transgenic plants were significantly higher than in WT plants. In response to Cd stress, the fresh weight of L1 and L3 line plants were 1.6 and 2.2-fold higher than in WT, respectively; similarly, the roots of L1 and L3 plants were 1.5 and 1.9-fold longer than in WT, respectively (Figures 4B,C). To examine the influence of *BnPCS1* on Cd accumulation in Arabidopsis, 28-day-old WT and transgenic Arabidopsis seedlings grown in vermiculite were irrigated with half-strength Hoagland nutrient solution supplement with 50 μM Cd and allowed to grow for 14 days under normal management of water and fertilizer conditions. The Cd content in roots and shoots of WT and transgenic plants were measured. There were no significantly differences of Cd content among WT and two transgenic lines (L1 and L3). However, the two transgenic lines showed significantly higher levels of Cd than WT plants in shoots. Cd content in shoots of L1 and L3 was 0.93 and 0.89 mg⋅kg^–1^, which was 1.43 and 1.37-fold higher than WT (0.65 mg⋅kg^–1^), respectively (Figure 4D). We also analyzed the translocation factor (TF) of Cd in WT, L1 and L3. TF is defined as the ratio of metal concentration in plant roots to shoots, which is an important tool used to assess a plant’s potential for phytoremediation purposes. The TF of L1 and L3 was 0.86 and 0.82, which was significantly higher than that of WT (0.61) (Figure 4E). The results indicated that overexpression of *BnPCS1* contributes to the translocation of Cd from roots to shootsin transgenic Arabidopsis plants.

### Lipid Peroxidation and Accumulation of H_2_O_2_

**FIGURE 5:**
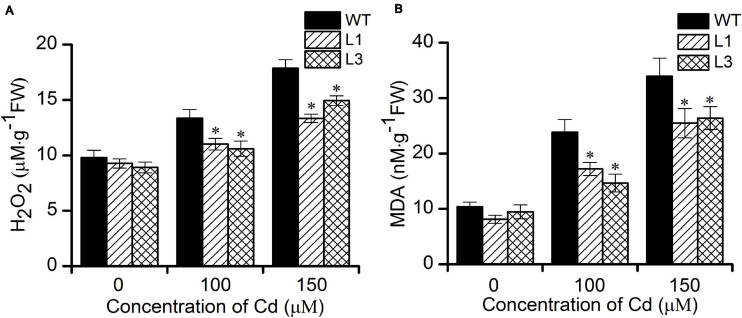
Cellular H_2_O_2_ and MDA contents in WT and *BnPCS1*-expressing transgenic Arabidopsis plantsin response to Cd stress. **(A)** H_2_O_2_ contents in WT and transgenic line plants treated with 0, 100,and 150 μM Cd for 2 days. **(B)** MDA contents in WT and transgenic line plants treated with 0, 100,and 150 μM Cd for 14 days. Data are presented as the means of three biological replicates with SE shown by vertical bars. Asterisks indicate significant differences (*p* < 0.05) in Cd-treated plants compared to WT.

It is well-known that heavy metal stress induces the production of reactive oxygen species (ROS) such as H_2_O_2_ and ${\text{O}}_{2}^{-.}$ which can inactivate enzymes and cause cellular damage by degrading proteins and interfering in pathways that are important in cell metabolism (Choudhury et al., 2013). MDA is one of the most well-known secondary products of lipid peroxidation, and it is thus often used as a marker for cell membrane injury (Grotto et al., 2009). To evaluate whether transgenic Arabidopsis have a higher antioxidative capability. We further measured the levels of H_2_O_2_ and MDA in WT and transgenic plants. As shown in Figures 5A,B, no significant differences in the H_2_O_2_ and MDA contents were found between the WT and transgenic plants. Following treatment with 100 and 150 μM Cd, the concentrations of H_2_O_2_ and MDA increased in all plants. However, plants of the transgenic lines L1 and L3 exhibited significantly lower levels of H_2_O_2_ and MDA than did the WT plants. These results show that overexpression of *BnPCS1* was able to reduce ROS production, which could potentially alleviate the oxidative damage to cells caused by Cd exposure in plants.

## Discussion

Previous studies have shown that the PC-dependent pathway is a key mechanism for resistance to heavy metals in plants (Kim et al., 2019). The production of PCs from glutathione is catalyzed by PCS, a protease-like enzyme. In the 30 years since the active PCS from *Silene cucubalus* was first isolated (Grill et al., 1989), nearly 100 kinds of *PCS* or*PCS*-related genes have been identified or predicted in plants, among which the *PCS* homologs from *Pteris vittata* (Dong, 2005), *Sesbania rostrata* (Li et al., 2009), *Triticum aestivum* (Couselo et al., 2010), tall fescue (Zhao et al., 2014) *Oryza sativa* (Das et al., 2017), and Ipomoea pes-caprae (Su et al., 2020) have been investigated and found to encode representative PCS proteins. There are single conserved domains at the N- and C-termini in all of these PCS proteins. The conserved N-terminal domain is thought to be a functional center of PCS catalytic activity. The conserved C-terminal domain of unknown function confers metal sensing ability and enzyme stability. In addition, the N-terminal region is more conserved than the C-terminal region (Yazdi et al., 2019; Hayashi et al., 2020). In this study, we isolated the full-length cDNA sequence of *BnPCS1* from *B. nivea*. Sequence alignment showed that BnPCS1 shares high deduced amino acid sequence identities with PCS proteins from other plant species. Two domains, phytochetatin and phytochelatin_C, were found to be present at the N- and C-termini of BnPCS1, respectively. Moreover, the N-terminal domain of BnPCS1 was more conserved than was the C-terminal domain (Figure 1A). The catalytic triad Cys^56^, His^162^, and Asp^180^ consists of three amino acid residues that are essential for the activity of PCS, and these are found in LjPCS1 (Ramos et al., 2007), AtPCS1 (Ha et al., 1999), and PcPCS1 (Hui et al., 2010). BnPCS1 also contains these three specific amino acids, which are Cys^58^, His^164^, and Asp^182^. Cys, as the only amino acid that possesses a sulfhydryl group among the 20 essential amino acids, can bind to heavy metal ions in different forms, which is considered to be important for the activation of PCS. For PCS, being rich in Cys is one of its important features. The C^358^C^359^XXXC^363^XXC^366^ motif is a heavy metal sensor, which is capable of transferring heavy metal ions to the N-terminal catalytic region when they are detected (Vestergaard et al., 2008; Li et al., 2020). This motif appears as C^368^C^369^RETC^373^MKC^376^ in LjPCS1 and C^369^C^370^QETC^374^VKC^377^ in PcPCS1, while this motif is present as C^371^C^372^QETC^376^VKC^379^ in BnPCS1 (Figure 1A), which is identical to the motif in PcPCS1 and similar to that in LjPCS1. These results reveal that BnPCS1 has similar characteristics to the PCS homologs from the other diverse plant species, and may perform similar functions.

Earlier studies suggested that *PCS* is a constitutively expressed gene and that there is no regulation of *PCS* at the transcriptional level in the PC synthesis process. Real-time PCR and Northern blotting results also showed that the transcriptional level of *AtPCS1* is not obviously influenced by Cd. Moreover, no significant difference in expression among the different tissues of Arabidopsis was observed (Vatamaniuk et al., 1999), and similar results were observed in tomato (Chen et al., 1997). However, more recent studies have shown that the expression of *PCS* can be regulated by heavy metal ions. The promoter of *AtPCS1* was fused to the *GUS* reporter gene and transformed into Arabidopsis, and GUS activity in transgenic Arabidopsis seedlings treated with Cd for 5 d increased 2-fold compared to that in untreated plants, although the difference gradually disappeared. This result indicates that the expression of *AtPCS1* is positively regulated early in plant development (Lee and Korban, 2002). Under Zn or Cd stress, the relative expression of two phytochelatin synthase genes from *Morus alba* was induced in root, stem and leaf tissues within 24 h of exposure to the metals, with Cd inducing expression more strongly than did Zn (Fan et al., 2018). The expression level of *SoPCS* were significantly unregulated in *Saccharum officinarum* roots under cadmium stress (Yousefi et al., 2018). The *MT2* and *PCS1* gene expression patterns in *Azolla* species were significantly induced by the heavy metal treatments (Cu, Zn, Ni, and Cd) (Talebi et al., 2019). In our study, the transcription of *BnPCS1* was upregulated significantly in the roots, stems, and leaves of *B. nivea* seedlings after they were treated with 100 μM Cd^2+^. This result indicates that the expression of *BnPCS1* can be induced by Cd treatment. The increased amount of *BnPCS1* mRNA in the leaves was significantly higher than in the roots and stems, which is similar to results for *PcPCS1* (Hui et al., 2010) and *NnPCS1* (Liu et al., 2012). The possible reasons for this are as follows: (1) *B. nivea* is a species of herbaceous perennial in the botanical family Urticaceae, and the replacement of the leaves occurs faster than the replacement of roots and stems; (2) the leaves of *B. nivea* are large with ample, well-developed vacuoles in the mesophyll cells, which can provide enoughbuffer space for Cd^2+^; (3) many trichomes are distributed on the leaf underside, and trichomes can accumulate heavy metal ions, including Cd^2+^ (Salt et al., 1995). Abscisic acid (ABA) and salicylic acid (SA) are two important plant hormones that are involved in signal transduction pathways, especially those that play roles in the responses of plants to a multitude of abiotic stresses. The expression of *StPCS1* in the roots of potato was strongly induced by exogenous ABA (Stroiński et al., 2010, 2013). Studies on the *PCS* genes in Arabidopsis showed that exogenous SA has no obvious influence on the transcription of *AtPCS1* and *AtPCS2* (Cazalé and Clemens, 2001). In this study, the expression of *BnPCS1* increased significantly when the *B. nivea* seedlings were treated with exogenous ABA, while no significant changes in the expression of *BnPCS1* were observed when the plants were treated with SA. These results indicate that the expression of *BnPCS1* may be independent of the SA signaling pathway, which is similar to the *PCS* genes from tomato and Arabidopsis.

It is generally accepted that PCS, which catalyzes PC synthesis as a catalytic enzyme, would be localized in the cytoplasm of cells. However, the subcellular localization analysis of BnPCS1 transiently expressed in Arabidopsis protoplasts showed that BnPCS1 was not only distributed in the cytoplasm, but also localized to the nucleus (Figure 2), which is similar to results of previous studies on AtPCS2, a PCS from Arabidopsis that is localized to both the cytoplasm and nucleus (Ding et al., 2013). This result indicates that BnPCS1 may participate in other special physiological processes. For example, some studies have shown that PCs may play important roles in maintaining homeostasis of nutrient ions such as Zn^2+^ (Tennstedt et al., 2009). Other studies have shown that PC-metal complexes, such as PCs-Cu and PCs-Zn, which function as metal ion donors for metal-dependent enzymes, can activate Cu^2+^ diamine oxidase (DAO; E.C.1.4.3.6.) and Zn^2+^ dependent carbonic anhydrase (CA; E.C.4.2.1) (Song et al., 2014).

The functions of PCS enzymes have been investigated through heterologous expression of *PCS* genes in prokaryotes, yeast, and model plants such as tobacco and Arabidopsis since they were first isolated from various species. However, the results from transgenic plants were various. Arabidopsis plants overexpressing *AtPCS1* showed hypersensitivity to Cd stress (Lee et al., 2003b) and enhanced As tolerance (Li et al., 2004), and the accumulation of Cd in the transgenic plants was decreased (Lee et al., 2003a). Heteroexpression of the wheat phytochelatin synthase gene (*TaPCS1*) in rice enhances cadmium sensitivity (Wang et al., 2012). On the contrary, expression of *AtPCS1* in Indian mustard enhanced its tolerance to As and Cd stress (Gasic and Korban, 2007). The accumulation of both As and Cd in tobacco was improved by overexpressing *CdPCS1*, a phytochelatin synthase gene from *Ceratophyllum demersum* (Shukla et al., 2012). The *OsPCS1* mutants of *Oryza sativa* exhibited increased sensitivity to As and Cd in hydroponic experiments, showing the importance of OsPCS1-dependent PC synthesis for rice As and Cd tolerance (Uraguchi et al., 2017). Tobacco expressing *NtPCS1* from *Nelumbo nucifera* exhibited an increased tolerance to As and Cd (Lee and Hwang, 2015). Overexpression of three duplicated *BnPCS* genes from *Brassica napus* enhanced Cd accumulation and translocationin *Arabidopsis thaliana AtPCS1* mutant (Bai et al., 2019). In our study, Arabidopsis seedlings overexpressing *BnPCS1* exhibited increased root lengths and fresh weights, but decreased H_2_O_2_ and MDA levels. Heavy metal stress can induce ROS such as H_2_O_2_ and ${\text{O}}_{2}^{-.}$, which can damage the plasma membrane through lipid peroxidation and other biomolecules such as DNA. MDA is one of the best known secondary products of lipid peroxidation, and it is often used as a marker of cell membrane damage. Compared to WT plants, lower H_2_O_2_ and MDA levels in *BnPCS1*-expressing transgenic Arabidopsis plants suggests that cellular damage was reduced. Cd accumulation in shoots and the translocation factor (TF) of transgenic lines were also found to be significantly higher than those of WT. TF is important for assessing the feasibility of a plant species for phytoremediation purposes. The increased TF indicated that *BnPCS1* can enhance the translocation of Cd from roots to shoots in plant. In conclusion, overexpression of *BnPCS1* confers enhanced Cd tolerance, accumulation and translocation in transgenic Arabidopsisplants, which could provide gene resources for phytoremediation.

## Conclusion

In this study, we isolated a phytochelatin synthase (PCS) gene, *BnPCS1*, from the bast fiber crop ramie (*Boehmeria nivea*). Sequence analysis indicated that *BnPCS1* encodes a protein of 56.02 kDa that is highly homologous to most of the PCS proteins reported from other plant species. The promoter of *BnPCS1* contains several cis-acting elements predicted to be involved in phytohormone signaling and a variety of stress responses. Subcellular localization analysis showed that BnPCS1 localizes to both nucleus and cytoplasm. Real-time PCR analysis showed that *BnPCS1* is significantly induced by Cd and ABA. Overexpression lines of *BnPCS1* exhibited better root growth and fresh weight, lower level of MDA and H_2_O_2_, and higher Cd accumulation and translocation factor compared to the WT under Cd stress. It will be of interest to explore the mechanism how *BnPCS1* is regulated by upstream factors, especially through the ABA-dependent pathways and further analysis in the distribution of PC-Cd complexes in plants.

## Data Availability Statement

The datasets presented in this study can be found in online repositories. The names of the repository/repositories and accession number(s) can be found in the article/Supplementary Material.

## Author Contributions

SZ designed the study and wrote the manuscript. WS and YJ performed the experiments. All authors discussed and interpreted the results.

## Conflict of Interest

The authors declare that the research was conducted in the absence of any commercial or financial relationships that could be construed as a potential conflict of interest.

## Footnotes

1 http://bioinformatics.psb.ugent.be/webtools/plantcare/html/

2 https://www.ncbi.nlm.nih.gov/orffinder/

3 https://web.expasy.org/protparam/

4 https://blast.ncbi.nlm.nih.gov/Blast.cgi

5 https://www.ncbi.nlm.nih.gov/Structure/cdd/wrpsb.cgi

6 https://www.genscript.com/wolf-psort.html

## Supplementary Material

Supplementary Figure 1 Nucleotide and deduced amino acid sequence of *BnPCS1* from *Boehmeria nivea.* Nucleotides are numbered on the left. The deduced amino acid residues are shown beneath the corresponding codons. An asterisk indicates the stop codon.

Supplementary Figure 2 Nucleotide sequence of the *BnPCS1* promoter region showing the cis-elements predicted by the PlantCARE database. The *BnPCS1* gene promoter sequence is 1,858 bp in length. The “A” of the ATG initiation codon is defined as +1. The CAAT-box, TATA-box, ABRE, ARE, CGTCA-motif, DRE1, G-box, STRE, TGACG-motif, and other important cis-regulatory elements are boxed and labeled.

Supplementary Figure 3 Kanamycin screening of *BnPCS1* transgenic *Arabidopsis thaliana* seedlings. T_0_-generation seeds of *Arabidopsis thaliana* plants transformed with the *BnPCS1* gene construct via the floral dip method were germinated on MS medium supplemented with 30 mg⋅L^–1^ kanamycin for screening. Transgenic lines remain green and grow, while the WT type seedlings stop growing and turn yellow.

Supplementary Figure 4 Identification of transgenic *BnPCS1 Arabidopsis thaliana* Col-0 seedlings by amplification of the *BsPCS1* gene using the primer pair PCS1-35S-F/PCS1-SP-R. The amplification products were electrophoresed on a 1.5% agarose gel at 5 volt/cm^2^, 1× TBE buffer for 0.5 h. M: Trans2K Plus II DNA size marker; P: positive plasmid control; CK: no template negative control; WT: Col-0 seedling; L1-L5: *BnPCS1* transgenic seedlings. The pBI121-BnPCS1 vector was used to transform *Arabidopsis thaliana* Col-0 plants via *Agrobacterium tumefaciens*-mediated transformation. All 35S::BnPCS1-overexpressing transgenic lines (T_1_ generation) were verified by PCR using the PCS1-35S-F and PCS1-SP-R primers to amplify *BcPCS1* from genomic DNA.

Supplementary Figure 5 Relative expression of the *BnPCS1* gene in transgenic *Arabidopsis thaliana* plants. WT: wild type Arabidopsis Col-0; L1-L5: *BnPCS1* transgenic *Arabidopsis* seedlings; Total RNA was extracted from leaves of transgenic plants overexpressing 35S:: *BnPCS1* (T_2_ generation) for qRT-PCR. *BnPCS1* transcript levels were significantly higher in two of the transgenic lines, L1 and L3, which were used in the experiments. Data are presented as the means of three biological replicates with SE shown by vertical bars.

Supplementary Table 1 List of oligonucleotide primers used in this study.

## Abbreviations

ABA: Abscisic acid
As: Arsenic
*B. nivea*: *Boehmeria nivea*
Cd: Cadmium
cDNA: Complementary DNA
Cu: Copper
DW: Dry weight
Fw: Fresh weight
GFP: Green fluorescent protein
GSH: Glutathione
ORF: Open reading frame
Pb: Lead
PCs: Phytochelatins
PCS: Phytochelatin synthase
qRT-PCR: Real-time quantitative polymerase chain reaction
RACE: Rapid amplification of cDNA ends
SA: Salicylic acid
TF: Translocation factor
WT: Wild-type
Zn: Zinc.
