# Supplementary material for: Overexpression of BnPCS1, a Novel Phytochelatin Synthase Gene From Ramie (Boehmeria nivea), Enhanced Cd Tolerance, Accumulation, and Translocation in Arabidopsis thaliana
Source: Front Plant Sci. 2021 Jun 15;12:639189. doi: 10.3389/fpls.2021.639189 (PMC8239399; doi:10.3389/fpls.2021.639189)
Supplement: Supplementary Figure 1 — Nucleotide and deduced amino acid sequence of BnPCS1 from Boehmeria nivea. Nucleotides are numbered on the left. The deduced amino acid residues are shown beneath the corresponding codons. An asterisk indicates the stop codon. [file Data_Sheet_1.zip › Supplementary Figure 1.DOCX]

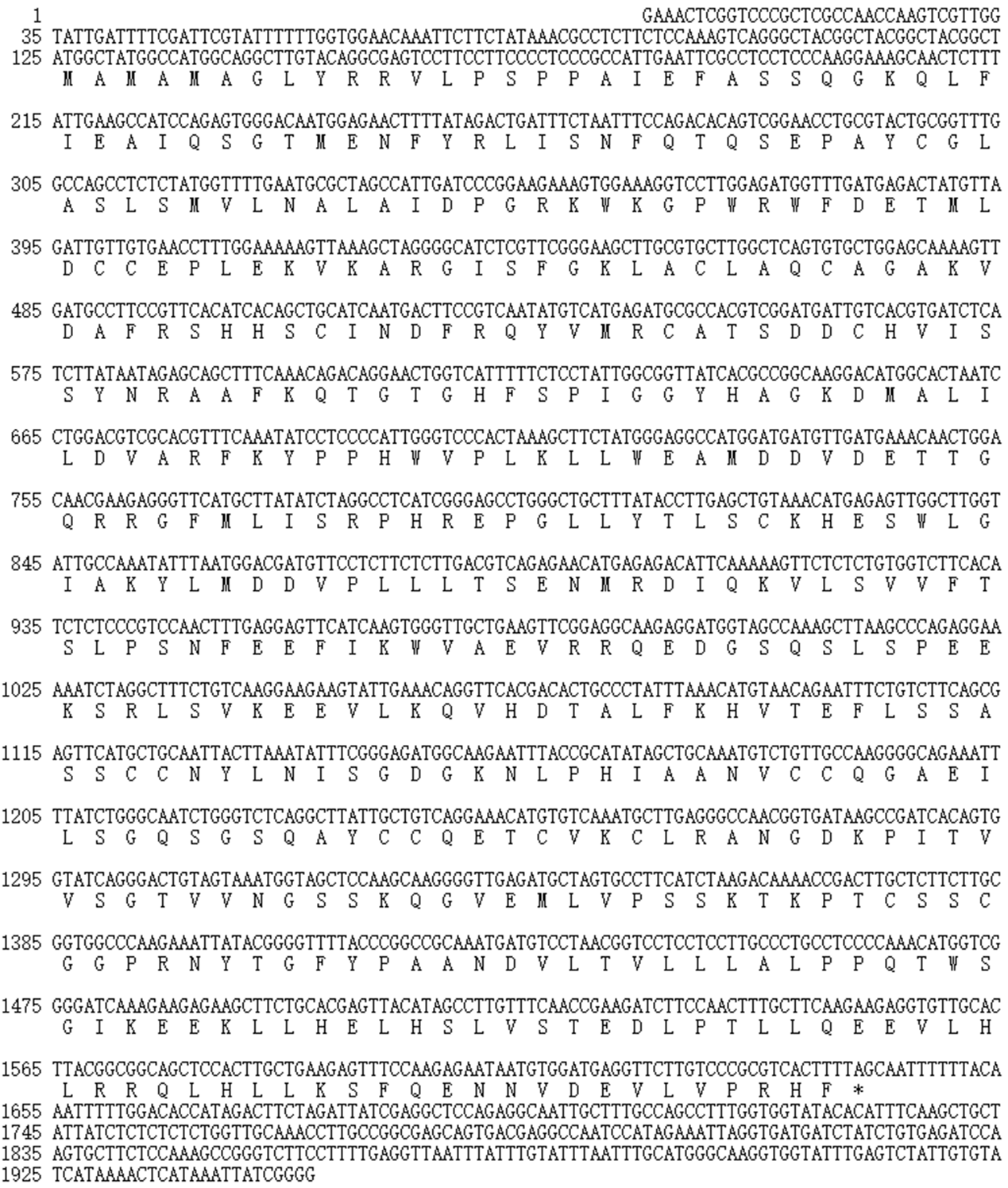


**Supplementary Figure 1. Nucleotide and deduced amino acid sequence of *BnPCS1* from *Boehmeria nivea.*** Nucleotides were numbered on the left. The deduced amino acid residues were showed under the corresponding codons. Asterisk indicates the stop codon.
