# Supplementary material for: Overexpression of BnPCS1, a Novel Phytochelatin Synthase Gene From Ramie (Boehmeria nivea), Enhanced Cd Tolerance, Accumulation, and Translocation in Arabidopsis thaliana
Source: Front Plant Sci. 2021 Jun 15;12:639189. doi: 10.3389/fpls.2021.639189 (PMC8239399; doi:10.3389/fpls.2021.639189)
Supplement: Supplementary Figure 1 — Nucleotide and deduced amino acid sequence of BnPCS1 from Boehmeria nivea. Nucleotides are numbered on the left. The deduced amino acid residues are shown beneath the corresponding codons. An asterisk indicates the stop codon. [file Data_Sheet_1.zip › Supplementary Figure 2.DOCX]

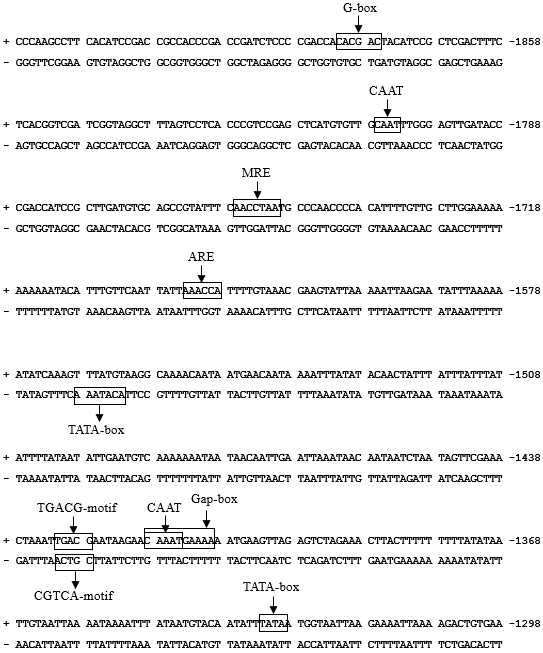

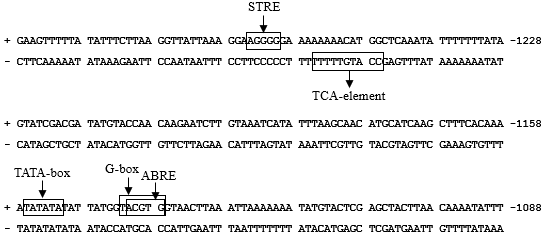


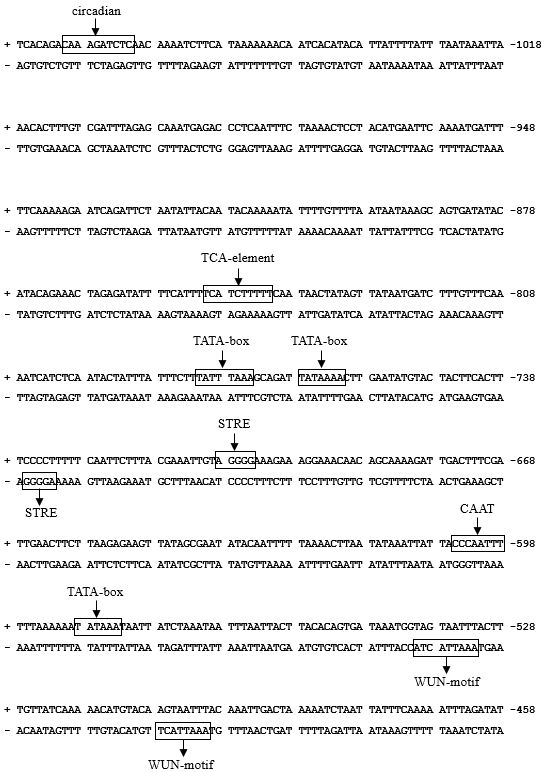

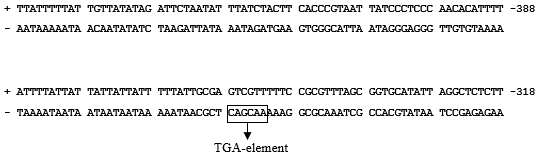


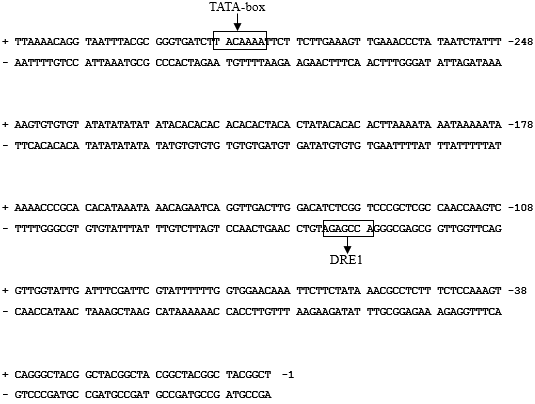


**Supplementary Figure 2. Sequence of *BnPCS1* promoter denoting the cis-elements predicted by PlantCARE database.** The length of *BnPCS1* gene promoter sequence was 1 928 bp. The A of the ATG initiation codon is defined as +1. The CAAT-box, TATA-box, ABRE, ARE, CGTCA-motif, DRE1, G-box, STRE, TGACG-motif and other important cis-regulatory elements are boxed and labeled.
