# Supplementary material for: Overexpression of BnPCS1, a Novel Phytochelatin Synthase Gene From Ramie (Boehmeria nivea), Enhanced Cd Tolerance, Accumulation, and Translocation in Arabidopsis thaliana
Source: Front Plant Sci. 2021 Jun 15;12:639189. doi: 10.3389/fpls.2021.639189 (PMC8239399; doi:10.3389/fpls.2021.639189)
Supplement: Supplementary Figure 1 — Nucleotide and deduced amino acid sequence of BnPCS1 from Boehmeria nivea. Nucleotides are numbered on the left. The deduced amino acid residues are shown beneath the corresponding codons. An asterisk indicates the stop codon. [file Data_Sheet_1.zip › Supplementary Figure 4.DOCX]

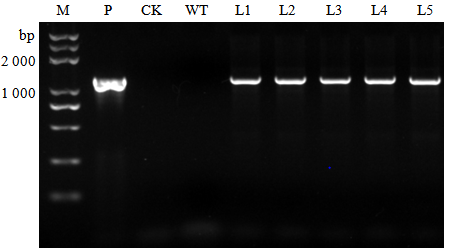


**Supplementary Figure 4. Identification of transgenic *BnPCS1* transgenic *Arabidopsis thaliana* seedlings by PCS1-35S-F/PCS1-SP-R primer.** M: Trans2K Plus II DNA Marker; P: positive plasmid control; CK: no template negative control; WT: common seedling; L1-L5: *BnPCS1* transgenic seedlings. The pBI121-BnPCS1 vectors were transferred by *Agrobacterium tumefaciens*-mediated genetic transformation into *Arabidopsis thaliana*. All overexpressing 35S::BnPCS1 transgenic lines (T1 generation) were verified by PCR using PCS1-35S-F and PCS1-SP-R primers.
