# Supplementary material for: Overexpression of BnPCS1, a Novel Phytochelatin Synthase Gene From Ramie (Boehmeria nivea), Enhanced Cd Tolerance, Accumulation, and Translocation in Arabidopsis thaliana
Source: Front Plant Sci. 2021 Jun 15;12:639189. doi: 10.3389/fpls.2021.639189 (PMC8239399; doi:10.3389/fpls.2021.639189)
Supplement: Supplementary Figure 1 — Nucleotide and deduced amino acid sequence of BnPCS1 from Boehmeria nivea. Nucleotides are numbered on the left. The deduced amino acid residues are shown beneath the corresponding codons. An asterisk indicates the stop codon. [file Data_Sheet_1.zip › Supplementary Figure 5.DOCX]

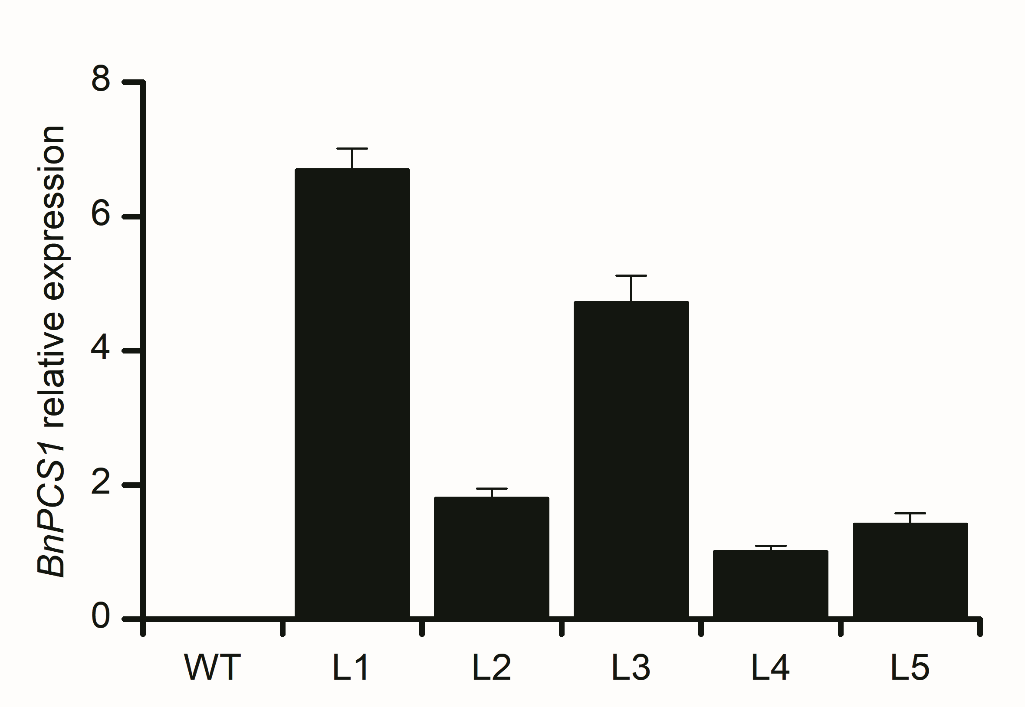


**Supplementary Figure 5. The relative expression of *BnPCS1* gene in transgenic *Arabidopsis thaliana***.

WT: wild type Arabidopsis; L1-L5: *BnPCS1* transgenic *Arabidopsis* seedlings; Total RNA were extracted from leaves of overexpressing 35S:: *BnPCS1* transgenic lines (T2 generation) for qRT-PCR. *BnPCS1* transcript levels were significantly high in several transgenic lines, the overexpression effect is excellent in L1 and L3 lines. Data are presented as the means of three biological replicates with SE shown by vertical bars.
